# Case report of a type III endoleak presenting only decades after endovascular aortic repair

**DOI:** 10.1016/j.ijscr.2019.01.008

**Published:** 2019-01-18

**Authors:** T.S.Q. Lee, T.T. Chong, J.C.C. Wang, T.C.E. Choke, T.Y. Tang

**Affiliations:** Department of Vascular Surgery, Singapore General Hospital, Singapore

**Keywords:** EVAR, Endoleak, Type III, Surveillance

## Abstract

Introduction: Type III endoleaks are a rare but potentially life-threatening complication post endovascular aortic aneurysm repair (EVAR).

Case report: A 91-year-old Chinese female, presented to our accident and emergency department for severe back and abdominal pain. She had previously undergone an EVAR procedure twenty years ago for a 6.5 cm diameter infra-renal abdominal aortic aneurysm. A CT aortogram revealed a type III endoleak, with the contralateral limb found to be disconnected from the main graft body. She was successfully treated by relining the graft using an endovascular technique.

Discussion: The case highlights the need for life-long stent-graft surveillance. We discuss early generation stent-grafts, type III endoleak treatment options and the current long-term data for late EVAR-related complications.

Conclusion: For patients who had undergone EVAR, type III endoleaks can present only decades later and pose a significant risk of aneurysmal rupture.

## Highlights

• Late type III endoleak is a rare but potentially life-threatening complication post endovascular aortic aneurysm repair.
• They can present only decades after surgery, even after an extended complication-free period.
• First line treatment often employs an endovascular approach to realign the endoleak with additional stent-grafts.
• Current long-term data for EVAR-related complications highlights the need for life-long stent-graft surveillance.

## 1 Introduction

Late type III endoleak is a rarely reported complication post endovascular aortic aneurysm repair (EVAR), with an estimated incidence of between 0.9–3% [1,2,6,8]. It is characterized by fabric tears or disruptions, component disconnections, or disintegration of the stent-graft [3]. The main concern of type III endoleaks is its risk of re-pressurizing the aneurysmal sac, leading to subsequent rupture. Indeed, type III endoleaks have been shown to be the pre-dominant cause of rupture and late conversion to open repair post EVAR, along with type I endoleaks [2,4]. As such, early intervention is often warranted once the endoleak is identified. We report a rare case of type III endoleak developing only two decades after endovascular aortic aneurysm repair.

This case has been reported in line with the SCARE Criteria [9].

## 2 Case presentation

A 91-year-old Chinese female with a background of hypertension and ischemic heart disease was admitted with a three-day history of central abdominal and back pain. She had previously undergone an endovascular aortic aneurysm repair (EVAR) twenty years prior, with a bifurcated endovascular aortic Vanguard device (*Boston Scientific Ltd, Marlborough, MA, US*) for a 6.5 cm diameter infra-renal abdominal aortic aneurysm. Until this admission, she was on regular stent-graft surveillance and had been free of any EVAR-related complications or re-interventions. Her blood pressure has been well controlled on a single agent – Amlodipine and takes only aspirin for secondary prevention for cardiovascular disease.

**Fig. 1:**
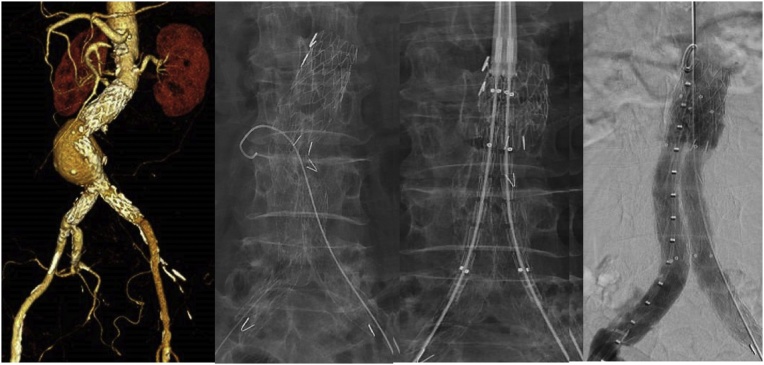
XXX.

A pre-operative CT aortogram (CTA) was performed and showed that the contralateral limb of her graft had become disconnected from the main body (type III endoleak), with an interval increase in size of the aneurysmal sac from 4.5 to 4.8 cm the past year. In view of the potential risk of aneurysmal rupture, the patient was counselled for and underwent percutaneous relining of the graft using two kissing Endurant^™^ limbs (*Medtronic Ltd, Dublin, Ireland).* This was performed by our consultant surgeon Mr Tang Tjun Yip. During the procedure, there was some anticipated difficulty cannulating the disconnected graft from the ipsilateral groin because of the angulation of the limb fracture and a contralateral wire from the main body side had to be snared first through the disconnected limb to get a through and through wire (see Fig. 1). Kissing stents had to be deployed to reposition the bifurcation of the graft; otherwise one side would have allowed one limb to hang over to the other side and potentially impinge on the blood flow to the other side, as the main body length of this graft is relatively short. The patient tolerated the procedure well and did not report further episodes of abdominal and back pain. She recovered well and was discharged two days post-procedure after a repeat CTA scan showed a no further endoleak. We reviewed her in clinic three months later, with a follow-up CTA showing no endoleaks, stable aneurysmal sac size and no narrowing of the limbs from the relining process.

## 3 Case discussion

Type III endoleaks can be divided into two subtypes [3]. Type IIIa endoleak originates from disconnection of the modular components as in this case. Type IIIb endoleak originates from a defect in the stent-graft fabric and it may be hard to detect with CTA and/or angiography, unless confirmed by open surgery. While the overall incidence of type III endoleaks have been reportedly low, early generation stent-grafts have been associated with higher rates of endoleaks [7]. The Vanguard aortic graft used in our patient belonged to this group of ‘early industry’ first generation stent grafts, which was introduced to market in the mid-1990s. One of the main concerns of the Vanguard aortic graft was its short main body. Due to its relative short stent overlap on the side of the contralateral limb, there was a higher risk of limb disconnection over time, due to kinking of the graft as the aneurysm sac shrunk [5]. This led to a number of type III endoleaks, and its withdrawal from the market in the late 1990s. Over time, successful generations of stent grafts have built upon previous experience and technological advancements, improving efficiency, outcomes and procedural success.

The treatment of late type III endoleak often employs an endovascular approach as first-line. Options include re-aligning the existing graft with bifurcated stent grafts or extension grafts to overlap the area of defect [6]. The procedures are technically straightforward, as long as the interventionist is able to negotiate the offset components and cannulate the main body gate, as in our case. Failing endovascular treatment, open repair can be considered. These include endograft preservation with in situ suturing, partial endograft explantation with reanastomosis or total explantation with replacement [1].

Given the introduction of EVAR only in the early 1990s in surgically high-risk patients, the long-term follow-up data in the literature are currently limited. In a meta-analysis of EVAR-1, DREAM, OVER and ACE trials, Powell et al. [8] reported AAA-related mortality to range from 5 to 11% of all-cause mortality more than four years post EVAR. For patients eight years post EVAR, aneurysm-related deaths, serious and life-threatening intervention rates were 1.4,1.6 and 2.1 per 100 person-years respectively, comparable with those for patients 6 months to 8 years post EVAR. As evident, the risks for long-term EVAR-related complications are not insignificant. As our patient demonstrated, even after a complication-free interval period of almost twenty years, the stent-graft stability should be not taken for granted. The case highlights once again the importance and need for lifelong aortic stent graft surveillance.

## 4 Conclusion

For patients who had undergone EVAR, type III endoleaks can present decades later and pose a significant risk of aneurysmal rupture. Once identified, early treatment is often warranted. While the improvements in successive generations of stent-grafts may have reduced rates of endoleaks, the current long-term data for patient outcomes post EVAR suggests the need for life-long aortic stent-graft surveillance.

## Conflict of interests

We declare no conflict of interest in this study.

## Funding

We declare no sponsors in funding.

## Ethical approval

This study did not require ethical approval as it was exempt in my institution.

## Consent

Patient has given explicit consent for the write-up of her case.

## Author contribution

Mr Terence Lee and Tang Tjun Yip contributed to the writing, and submission of the paper.

Mr Tang Tjun Yip and Chong Tze Tec were directly involved with the patient’s diagnosis, treatment and care.

Mr John Wang, Chong Tze Tec and Edward Choke critically appraised and reviewed the paper.

## Registration of research studies

This study is not registered.

## Guarantor

Mr Tang Tjun Yip.

## Provenance and peer review

Not commissioned, externally peer-reviewed
